# Using Imputation to Provide Location Information for Nongeocoded Addresses

**DOI:** 10.1371/journal.pone.0008998

**Published:** 2010-02-10

**Authors:** Frank C. Curriero, Martin Kulldorff, Francis P. Boscoe, Ann C. Klassen

**Affiliations:** 1 Department of Environmental Health Sciences and Department of Biostatistics, Johns Hopkins Bloomberg School of Public Health, Baltimore, Maryland, United States of America; 2 Department of Population Medicine, Harvard Medical School and Harvard Pilgrim Health Care Institute, Boston, Massachusetts, United States of America; 3 New York State Cancer Registry, New York State Department of Health, Albany, New York, United States of America; 4 Department of Health, Behavior, and Society, Johns Hopkins Bloomberg School of Public Health, and Department of Oncology, Johns Hopkins School of Medicine, Baltimore, Maryland, United States of America; University of Swansea, United Kingdom

## Abstract

**Background:**

The importance of geography as a source of variation in health research continues to receive sustained attention in the literature. The inclusion of geographic information in such research often begins by adding data to a map which is predicated by some knowledge of location. A precise level of spatial information is conventionally achieved through geocoding, the geographic information system (GIS) process of translating mailing address information to coordinates on a map. The geocoding process is not without its limitations, though, since there is always a percentage of addresses which cannot be converted successfully (nongeocodable). This raises concerns regarding bias since traditionally the practice has been to exclude nongeocoded data records from analysis.

**Methodology/Principal Findings:**

In this manuscript we develop and evaluate a set of imputation strategies for dealing with missing spatial information from nongeocoded addresses. The strategies are developed assuming a known zip code with increasing use of collateral information, namely the spatial distribution of the population at risk. Strategies are evaluated using prostate cancer data obtained from the Maryland Cancer Registry. We consider total case enumerations at the Census county, tract, and block group level as the outcome of interest when applying and evaluating the methods. Multiple imputation is used to provide estimated total case counts based on complete data (geocodes plus imputed nongeocodes) with a measure of uncertainty. Results indicate that the imputation strategy based on using available population-based age, gender, and race information performed the best overall at the county, tract, and block group levels.

**Conclusions/Significance:**

The procedure allows for the potentially biased and likely under reported outcome, case enumerations based on only the geocoded records, to be presented with a statistically adjusted count (imputed count) with a measure of uncertainty that are based on all the case data, the geocodes and imputed nongeocodes. Similar strategies can be applied in other analysis settings.

## Introduction

The growing recognition of the importance of geography to areas of public health research and practice, such as cancer control science, planning, and service delivery, has led to increased efforts on the part of State and local health authorities to add geographic location information to surveillance and service data [1]–[4]. The most precise level of location information (outside of the use of a global positioning system (GPS) device or procedures based on aerial imagery) is conventionally achieved through geocoding, the geographic information system (GIS) process of translating mailing address information to longitude and latitude coordinates on a map.

Fundamentally speaking, geocoding is a means to add data on a map. Once successfully mapped, the data locations can provide gateway access to a plethora of opportunities for geographically linking to other sources of information. This holds regardless of whether the geocoded data element is a cancer outcome or other health or non-health related outcome [5]–[7]. Supporting data layers of interest may include environmental data such as air, soil, and water parameters; socio-economic and demographic data, such as the common information accessible through the US Census; the Census administrative and other geographic boundaries; as well as information from the built environment; such as hospitals, locations of health care providers, and other point sources. Mapped data also allows for distances to be measured and proximity characterized, both of which may be of interest.

From an analysis perspective, a primary reason for mapping data and linking it to other geographic data sources is to include a level of spatial variation (or geographic variation) regarding an outcome of interest. Even when spatial variation may not be of intrinsic interest, data that are spatially linked often exhibit spatial auto-correlation, a property that would need to be adjusted for in standard statistical procedures that assume independence [8]. Spatial Analysis in public health can usually be categorized as those exploring clustering, cluster detection, and/or spatial variation in outcome risk [9]–[19]. Other analysis might focus more on disease mapping applications [20]–[22], an example of which would include enumeration of cases or rates aggregated to some of the common Census administrative units. These examples, which by no means is an exhaustive list, are indicative of the continued use of mapped data in the public health arena.

Adding information to a map via the geocoding process is not without its limitations, though, since there is always a percentage of addresses which cannot be converted successfully (nongeocodable). Reasons for unsuccessful geocoding include address error, base maps out of date, and addresses that are a PO Box or rural postal route. The practice and details of geocoding has received recent attention especially in the health research literature [4]. There are now references to guidelines on geocoding with efforts designed to increase what has been termed the geocoding hit or match rate, the proportion of successfully geocoded records for a given data set [23]–[25].

When dealing with nongeocoded data, traditionally the common practice has been to geocode as best as possible and then have analysis or mapped descriptions proceed just based on those that geocoded successfully. In general, analysis and interpretations based on incomplete data raises issues regarding bias. In the current setting additional concerns regarding spatial selection bias may be raised when excluding nongeocoded data due to the fact that geocoding success, more precisely the prevalence of nongeocodes, is often not geographically neutral [26], [27]. It is not uncommon for rural area addresses to be more susceptible to unsuccessful geocoding than those addresses located in more urban areas [28]–[31]. An alternative practice could be to analyze the data at a higher level of aggregation, such as with zip codes which are most often reported with address information. This leads to a loss of spatial resolution as well as other potential drawbacks (e.g. ecological bias) even if all cases can be retained. Therefore, it is always preferable to base analyses on the most detailed location information available, although when using highly confidential data, it may be necessary to report or display results in a more aggregated format.

In this manuscript we develop and evaluate a set of imputation strategies for dealing with nongeocoded addresses so that analysis can proceed using all case records. The practice of imputation is a common statistical tool used to fill in missing data values. Little and Rubin 2002 [32] provide a comprehensive treatment of imputation and its applications. Imputation for nongeocoded addresses involves assigning longitude and latitude coordinates or some other appropriate geographic identifier so that analysis for complete data can be applied. We devise three strategies for imputing location information for nongeocoded addresses with increasing use of available information. Versions of these strategies were originally developed and applied by Klassen et al. 2004 [11] and are similar to those presented by Boscoe 2008 [25] and Henry and Boscoe 2008 [33], see also Zimmerman 2008 [34] for related discussions. New in this manuscript is the inclusion of a measure of imputation uncertainty and an evaluation procedure based on a relevant analysis outcome.

The imputation strategies are developed and evaluated using prostate cancer data obtained from the Maryland Cancer Registry. Case enumerations at the Census county, tract, and block group level are considered as the outcome of interest when applying and evaluating the imputation methods. Multiple imputation is used to provide estimated total case counts based on complete data (geocodes plus imputed nongeocodes) with a measure of uncertainty. The procedure allows for the potentially biased and likely under reported outcome, case enumerations based on only the geocoded records, to be presented with imputed statistically adjusted results.

## Methods

### Maryland Prostate Cancer Data

Data for these analyses are part of a larger data set used to investigate geographic patterns of prostate cancer burden in Maryland, based on all incident cases of prostate cancer reported to the Maryland Cancer Registry during 1992–1997 (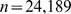). Data were obtained under a data use agreement between the Maryland Department of Health and Mental Hygiene and the researchers, with approval from the institutional review boards of the Johns Hopkins Bloomberg School of Public Health and the Maryland Department of Health and Mental Hygiene. Findings related to prostate cancer outcomes have been reported previously, including area-level predictors of prostate cancer stage at diagnosis and tumor histologic grade [11], predictors of missing data on staging and grade in registry cases [12], and geographic clustering of tumor and other prostate cancer characteristics [15], [35]–[36].

We determined that 23,993 cases had verifiable Maryland addresses. Using each case's reported address at diagnosis, we geocoded all cases in ArcGIS [37] to longitude and latitude coordinates, using supplemental commercially available address cleaning software and three base maps for Maryland to maximize matching [23], resulting in 21,904 geocoded cases, a geocoding match rate of 91%. Analysis of these data cited above [11]–[12], [15] have utilized the imputation algorithm described below (Strategy 3) to assign location to the remaining 2089 cases, and minimize bias from missing cases.

For the purposes of the current exercise, we utilized a subset of the entire data set, comprised of all geocoded cases with complete information on age, race, stage and grade (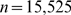). This reduced data set is appropriate for developing and evaluating the proposed imputation strategies.

Demographic data including age (in Census 5 year categories), race (black or white), and gender population counts for Maryland were obtained from the 1990 US Census Summary File 1 (SF 1) for the Census geographic units beginning at the Census block (the smallest geographic unit for which the Census Bureau tabulates data), block group, tract, and county [38]. Census geography follows a hierarchical structure with blocks nested within block groups, block groups nested within tracts, and tracts nested within counties. Boundary files for all Census geography were also obtained and used in the evaluation phase for imputing nongeocoded addresses. There are 24 counties, 1151 tracts, and 3670 block groups defined in the 1990 US Census for Maryland [38], all of which are represented in the 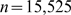 data subset used in our analysis.

### Imputation Strategies

The strategies proposed assign nongeocoded addresses to a Census block, block group, tract, and county based on an assumed correctly reported zip code. If desired, longitude and latitude coordinates can be taken as a center point of the assigned block (e.g. the geographic center or population center) or a randomly chosen point within that block, since Census blocks are the smallest units considered.

Note that zip codes are US postal units and do not generally coincide spatially with the Census hierarchy of geographic units. For all strategies considered, Census blocks were designated to be within a zip code if their geographic center fell within the zip code boundary. Block groups, tracts, and counties containing a block designated to a zip code were then also considered associated with that zip code. Since these Census units contain multiple blocks, it is possible for them to be associated with more than one zip code.

As a side note, in 2000 the US Census introduced Zip Code Tabulation Areas (ZCTAs) as a new geographic unit. ZCTAs are composed of Census blocks and therefore spatially coincides with the Census hierarchy of geographic units while also closely matching spatially with US postal zip codes [39]. As our analysis here is based on 1990 Census data, it does not involve the use of ZCTAs.

**Figure 1:**
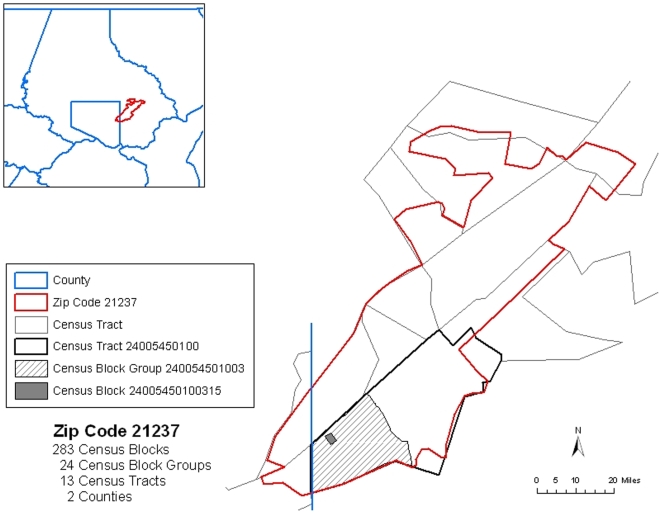
The 1990 Census geography for Maryland zip code 21237. Shown are the 13 Census tracts and 2 counties (Baltimore County and Baltimore City) associated with zip code 21237. Census tract 24005450100 is highlighted with one of its block groups (240054501003) and one of its blocks (24005450100315) identified.

For discussion below consider a fictitious nongeocoded Maryland prostate cancer case: a white male between the age of 45 and 49 residing in Maryland zip code 21237. From the 1990 US Census there are 283 Census blocks, 24 block groups, 13 tracts, and 2 counties associated with zip code 21237. Figure 1 displays the 1990 Census geography for zip code 21237 showing these 13 tracts and 2 counties. The finer block group and block subdivisions are shown in Figure 1 for a selected Census tract and block group.

#### Imputation Strategy 1

Imputation Strategy 1 assigns a nongeocoded address to a randomly selected Census block, block group, tract, and county within the known zip code. This simplistic approach uses only the zip code information and provides a baseline for developing more sophisticated methods based on other known information. In regards to the above nongeocoded case example a Census block, block group, tract, and county within zip code 21237 would be randomly selected as the imputed location. For example, imputation at the Census tract level would randomly select one of the 13 tracts associated with zip code 21237 (Figure 1). Each tract has equal probability of being selected under Strategy 1 and the weight 1/13 also represents the probability of imputing to the correct Census tract for a given nongeocoded address. The selection probabilities for the other Census units under Strategy 1 are 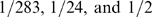 respectively for imputation to Census blocks, block groups, and counties within zip code 21237. As with tracts these weights also represent the probability of imputing to the correct corresponding Census unit for a given nongeocoded address using Strategy 1.

#### Imputation Strategy 2

Imputation Strategy 2 first assigns a nongeocoded address to a randomly selected Census block within the known zip code. Assignment to the other units are then based on converting the selected Census block to its encompassing block group, tract, and county, by exploiting the nested hierarchy of the Census geography. Strategies 1 and 2 are equivalent in regards to Census block imputation, but assignment to the other units are weighted based on the number of Census blocks they contain, with higher weight given to those units that contain more blocks. In comparison to the random imputation for all Census units in Strategy 1 this Census geography based weighting scheme used for Strategy 2 may better reflect population density.

In regards to the nongeocoded case example a Census block in zip code 21237 would be randomly selected (out of the 283 contained in zip code 21237) as the imputed block assignment. Block identifiers employed by the US Census in 1990 are comprised of a fourteen digit string of numeric characters with an occasional 15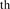 alphabetic character included on the right. Converting a block identifier to its encompassing block group, tract, and county identifiers can be accomplished by retaining the first twelve, eleven, and five left hand digits respectively, providing a straight forward and feasible computational approach. The first two digits are the Census state identifiers, 24 for Maryland. For example, suppose the Census block selected was the block with Census ID 24005450100315. The encompassing Census block group, tract, and county, that contains this block, would be identified with the IDs 240054501003, 24005450100, and 24005 respectively. This Census block, block group, and tract are shown highlighted in Figure 1.

With focus on imputation to the Census tract level, the distribution of the number of blocks contained in each of the 13 tracts in zip code 21237 range between 1 and 89 blocks with larger number of blocks per tract suggestive of higher population density. Following the previous example, Census tract 24005450100 contains the largest number of blocks (89) and therefore has the highest probability of being selected. The probability of selecting this tract for imputation with Strategy 2 is thus given by 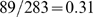 in comparison to the selection weight of 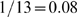 for this tract under Strategy 1. Similar procedures were applied for imputing to Census block groups and counties under Strategy 2.

#### Imputation Strategy 3

Imputation Strategy 3 assigns a nongeocoded addresses to a Census block within their known zip code using a weighted probability of assignment according to the spatial distribution of the available population. Continuing with this same example, Census demographic data can be used to estimate the expected number of white males between the age of 45 and 49 for all blocks in zip code 21237. A block would then be randomly selected using the ratio of block expected counts to the corresponding zip code total
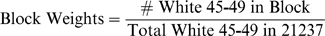
as the selection probabilities. In comparison, Strategies 1 and 2 randomly selected blocks with equal weight (of 1/283 for zip code 21237). Assignment to the other units for Strategy 3 follow as in Strategy 2, converting the selected Census block to its encompassing block group, tract, and county which by the way is equivalent to probability assignments based on these respective summed block weights for each of these units.

The 1990 US Census does not release tabulated age by gender by race specific information at the block level. Expected block level counts for males of a specific age and race category were estimated by multiplying the Census-provided block level racial total by the corresponding block group level age and race rate. That is, by applying the block group level proportions in each age/race strata to those at the block level for a given race.

Prior to imputing any nongeocoded cases, information from successfully geocoded cases were used to calculate adjusted population totals for Strategy 3. For example, using the above scenario, the number of white male cases between the age of 45 and 49 that have been successfully geocoded to a location within zip code 21237 (for which their block can easily be identified) are subtracted from the corresponding expected block counts, yielding an adjusted expected count more accurately reflecting the availability of the remaining population strata. Selection probabilities and imputation then follow as described. If a block contained no Census population in a given case's specific race and age group category, before or after adjusting for the successfully geocoded cases, the probability of assignment to that block was set to zero. In a further adjustment that was not explored here, once a nongeocoded case has been imputed it can be added to the pool of geocoded cases so the adjusted population strata can be continually updated.

### Imputation Performance Evaluation

For experimental purposes we use the reduced geocoded Maryland prostate cancer data (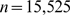 cases) and consider those records that geocoded with an ArcGIS address match score of 100% as the *experimental geocoded* subset (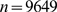 cases) and those that geocoded with an address match score less than 100% as the *experimental nongeocoded* subset (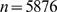 cases). Addresses that geocoded in ArcGIS are assigned a match score (ranging from 0 to 100) as a description of how well each address element (e.g. range of street number, spelling of street name, missing or incorrect street direction) matched the information in the available base maps [37]. Dividing the data set in this manner provided an alternative to simple random subsets and perhaps yielded a more representative set of what might approximate nongeocoded addresses. Additionally, the high percentage of experimental nongeocoded cases (38% nongeocode, 62% geocode) provided more information to better evaluate the properties of the imputation strategies at geographic units smaller than county.

As a benchmark for evaluation we consider the total number of enumerated cases per Census unit as an outcome of interest which is known for the geocoded data set (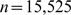). The imputation strategies defined previously were applied to the *experimental nongeocode* cases resulting in a set of imputed Census units for each case record. These imputed cases were then combined with the *experimental geocodes* providing a complete data set (geocoded plus imputed nongeocodes) for which case enumerations can be calculated and compared to that obtained using the complete geocoded data set. Performance of the imputation process was evaluated using multiple imputation as outlined below [32].

1. Impute the experimental nongeocode cases using Strategies 1, 2, and 3.
2. Combine the imputed experimental nongeocode results from each strategy with the experimental geocodes yielding complete data sets.
3. Enumerate case counts for each Census unit using the imputed complete data sets.
4. Steps 1–3 were repeated 1000 times.

This algorithm yields, for each strategy and Census unit (block group, tract, and county), a distribution of 1000 imputed enumerated case counts. Results are summarized by considering the mean and the middle 95%, taken as 2.5 and 97.5 percentiles, of these distributions as the estimated imputed count and measure of uncertainty respectively. The 95% multiple imputation interval reflects variability in the range of potential imputed results and was considered informative for the current application as well as being independent of the number of multiple imputations performed. Alternatively, uncertainty in imputed results could have been characterized with multiple imputation-based confidence intervals around the mean imputed totals [32]. These, however, yielded artificially narrow intervals due to the number (1000) of multiple imputations performed. If all case records in the relevant zip code were geocoded successfully (and assumed correctly), the total enumerated cases per Census unit in that zip code would be taken as a fixed non-random quantity. Imputation uncertainty is therefore based on the distribution of imputed case totals across the zip code.

Alternatively, imputation performance could be evaluated on the individual case level, identifying for example, whether each experimental nongeocoded case was imputed to its correct Census unit. This was essentially the approach used in Henry and Boscoe 2008 [33], applying a multiple imputation approach to estimate expected rates of imputing to the correct Census units. As pointed out earlier geocoding is more a means to an end by providing a link to other sources of spatial information supporting further analysis. Knowledge of the imputation success rate for any individual or collection of nongeocoded case records (although informative) is therefore limited in such endeavors when that success rate does not infer any performance related properties for how the geocoding and imputed nongeocodes are used in analysis. This was the motivation behind our consideration to use the simple case enumeration outcome per Census unit. Further, imputation evaluated at the individual case level is essentially independent Bernoulli trials. The expected success rates and their variance can be determined analytically without the need for multiple imputation since the weights (i.e. probability of imputing to the correct Census units) are known for all strategies in this type of experiment where we know the truth, but subset the data to behave as though we do not. The assumption of independence, however, is clearly no longer justified when an outcome of the geocoding process that is a function of more than one case is considered, such as with case enumerations.

The imputation strategies and multiple imputation based performance evaluation analysis were coded and performed in the R Statistical Computing Environment [40].

## Results

**Figure 2:**
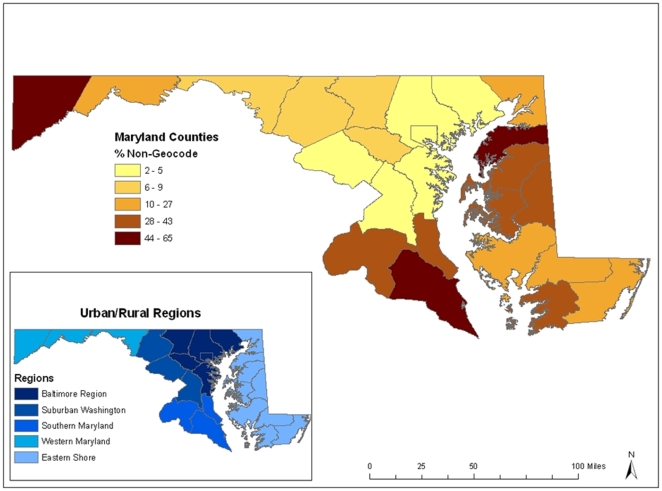
Maryland county level percent nongeocodes and urban/rural status. Maryland county level percent of nongeocoded case records (ratio of nongeocoded cases to the total nongeocoded plus geocoded cases) based on the geocoded 1992–1997 Maryland prostate cancer data subset (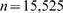). Also shown on the insert map is the 1990 US Census county level urban/rural categorization from the most urban (Baltimore Region) to most rural (Eastern Shore).

Figure 2 displays, for the geocoded subset of the 1992–1997 Maryland prostate cancer data (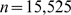) used in this analysis, the county level percent of nongeocoded case records (the ratio of nongeocoded cases to the total nongeocoded plus geocoded cases). Based on 1990 US Census population data the 24 Maryland counties can be categorized by geographic region from most urban (Baltimore Region) to most rural (Eastern Shore). These regions are shown on the insert map in Figure 2. Nongeocoded data are often not geographically uniform and commonly related at least in part to population density with rural areas being more susceptible. Both these points are conveyed in Figure 2, highlighting the caution against the practice of removing nongeocoded data from analysis [26].

Results to follow evaluating the imputation strategies are based on this geocoded Maryland prostate cancer data subset (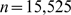 cases) and as described previously split into the experimental geocoded set (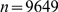 cases) and the experimental nongeocoded set (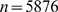 cases). To clarify, this data set has been completely geocoded according to the process outlined previously and for purposes here assumed geocoded correctly. Thus the Census block, block group, tract, and county are known for both the experimental geocoded and experimental nongeocoded data sets. Imputation strategies are applied to the experimental nongeocoded data as though this information was unknown as it would be for real nongeocoded data. Results from imputing the experimental nongeocoded data set are then combined with the experimental geocoded data, case totals for each Census unit calculated, and compared to the known totals.

Table 1 contains the geocoding results presented at the county level listed by geographic region. Specific counties within each region remain unidentified to protect case confidentiality in counties with small numbers of cases. For each county the total number of cases derived from the geocoded data set, taken in this experiment as the true, are followed by the number of experimental geocodes and experimental nongeocodes. For analysis intended to reveal geographic variation at units other than zip code, excluding nongeocoded case data will always lead to under-reporting. This is certainly clear in Table 1, though exaggerated since our experimental data set was formed to have only a 62% geocoding match rate.

**Table 1:** Imputation results for Strategy 3 at the county level stratified by urban/rural geographic region.

Geographic	Case Enumerations				Imputation	Ratio
Region	True	Geocoded	Nongeocoded	Imputed	Interval	Imputed/True
Baltimore Region						
	468	246	222	472.2	(464, 480) 	1.01
	475	288	187	472.5	(444, 481) 	0.99
	583	341	242	586.4	(580, 592) 	1.01
	1344	735	609	1340.2	(1333, 1347) 	1.00
	2788	2001	787	2824.9	(2806, 2843)	1.01
	3081	2032	1049	3044.6	(3025, 3064)	0.99
Suburban Washington						
	388	187	201	389.7	(383, 397) 	1.00
	1880	1241	639	1879.8	(1875, 1885) 	1.00
	2366	1579	787	2363.9	(2359, 2368) 	1.00
Southern Maryland						
	62	16	46	63.3	(61, 65) 	1.02
	117	54	63	115.9	(113, 118) 	0.99
	185	71	114	184.7	(182, 187) 	1.00
Western Maryland						
	38	6	32	38.0	(36, 41) 	1.00
	279	154	125	279.0	(276, 281) 	1.00
	356	222	134	355.2	(352, 358) 	1.00
Eastern Shore						
	42	12	30	37.3	(33, 41)	0.89
	51	3	48	49.9	(47, 52) 	0.98
	88	20	68	88.0	(87, 88) 	1.00
	97	49	48	101.8	(98, 106)	1.05
	139	96	43	139.0	(139, 139) 	1.00
	158	89	69	158.0	(158, 158) 	1.00
	160	38	122	160.0	(160, 160) 	1.00
	185	79	106	186.9	(184, 189) 	1.01
	195	90	105	195.0	(195, 195) 	1.00

Presented for each of the 24 Maryland counties are True total case enumerations with corresponding totals derived from those that were labeled as geocoded and those that were labeled as nongeocoded followed by an imputed total and a 95% multiple imputation interval computed using Strategy 3. Units for all results are number of cases. Imputation intervals are starred when they contained the true total. The ratio of imputed number cases to the true number cases is also listed. Results based on the 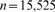 geocoded Maryland Prostate cancer data split into the 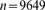 experimental geocodes and 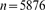 experimental nongeocodes subsets for evaluation.

Results from Imputation Strategy 3 are also summarized in Table 1 for each county by listing the average (expected) number of imputed cases and the 95% multiple imputation interval. Results are further highlighted as to whether the multiple imputation interval covered the true number of cases. For example, in our experiment the first county listed under the Baltimore Region had a true number of cases equal to 468. However, 222 of these cases fell into the experimental nongeocode category. Imputation Strategy 3 yielded an imputed number of cases of 472.2 with the 95% multiple imputation interval of 464 to 480 cases capturing the true total.

In addition to avoiding reporting the total number of geocoded cases (246 cases) as the final count, as has been done in practice, a benefit of this approach is that it provides a statistically adjusted count (i.e. imputed count) with a measure of uncertainty. For example, in this county a total count of 246 cases based on the geocoded records could be accompanied with an estimated count of 472.2 cases based on all records using imputation. Furthermore, the expected range of imputed counts for this county is 464 to 480 cases.

The ratio of imputed number cases to the true number cases is also listed in Table 1 to characterize imputation accuracy on a common scale. As is evident, most imputed counts were very close to their true values with a mix of those that were over and underestimated, ratios above or below 1.00 respectively. No trends across the five geographic regions were apparent, although some of the largest errors occurred in rural Eastern Shore counties. Note that for some counties the listed ratio of 1.00 was due to rounding.

All three imputation strategies were performed and evaluated at the Census county, tract, and block group level. Table 2 summarizes these results listing the proportion of imputation intervals that contained the true total as well as the average width of these intervals (in units of case counts), each computed over the 24 counties, 1151 tracts, and 3670 block groups that were represented in the geocoded data set. The 83.3% covered (20 of the 24 counties) with an average interval width of 9.4 for Strategy 3 at the county level was shown in more detail in Table 1. Evident from these results is that imputation for case enumerations at the county, tract, and block group level improves as we move towards Strategy 3 both in terms of the percentage of times the imputation interval contained the true total as well as average width of the intervals.

**Table 2:** Results for Imputation Strategies 1, 2, and 3 at the Census county, tract, and block group level.

	Imputation Approach		
Spatial Scale	Strategy 1	Strategy 2	Strategy 3
**County**			
% Covered	25.0%	83.3%	83.3%
Avg Interval Width	15.9	10.0	9.4
**Tract**			
% Covered	80.0%	86.2%	90.5%
Avg Interval Width	7.5	6.9	6.7
**Block Group**			
% Covered	93.7%	94.0%	95.8%
Avg Interval Width	4.1	3.9	3.8

Presented are the percentage of times the 95% multiple imputation intervals contained the true total case enumerations and the width of the imputation based intervals (in units of number of cases), averaged across the 24 Maryland counties, 1151 tracts, and 3670 block groups. Results based on the 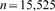 geocoded Maryland Prostate cancer data split into the 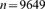 experimental geocodes and 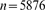 experimental nongeocodes subsets for evaluation.

Two interesting features of the Table 2 results warrant further mention. First, the width of the multiple imputation intervals are related to the total number of cases, those geocoded plus those in need of imputation. Interval widths are therefore wider on average for more populated regions and/or for larger Census units. Hence comparisons between multiple imputation interval widths are made only across the three strategies within a given Census unit. Second, also evident is what appears to be an advantage for better imputation success, in terms of the percentage of times the imputation intervals covered the true totals, for the smaller Census units, block groups. The larger variability for smaller sample sizes such as those experienced at the block group level actually produces wider multiple imputation intervals relative to the total enumerated cases than those at the larger units such as county. This, in combination with the fact that the total enumerated cases outcome is bounded below by zero, imparts greater odds of successfully covering with the multiple imputation interval at the smaller Census units as shown.

To provide information on geographic variation, the tract level results shown in Table 2 were stratified according to their geographic region and are presented in Table 3. Tract level results across geographic region are consistent with those contained in Table 2, imputations tend to improve moving toward Strategy 3 both in terms of the percentage of times the imputation interval contained the true total as well as average width of the intervals.

**Table 3:** Results for Imputation Strategies 1, 2, and 3 at the Census tract level stratified by urban/rural geographic region.

Geographic	Tract Level Imputation		
Region	Strategy 1	Strategy 2	Strategy 3
Baltimore Region			
% Covered	80.9%	86.3%	92.5%
Avg Width	7.9	7.4	7.2
Suburban Washington			
% Covered	82.5%	84.9%	90.1%
Avg Width	7.1	6.7	6.5
Southern Maryland			
% Covered	84.8%	93.5%	91.3%
Avg Width	6.5	5.5	5.0
Western Maryland			
% Covered	78.7%	83.6%	85.2%
Avg Width	7.4	6.6	6.5
Eastern Shore			
% Covered	65.7%	89.2%	85.2%
Avg Width	7.0	5.9	5.5

Presented are the percentage of times the 95% multiple imputation intervals contained the true total case enumerations and the width of the imputation based intervals (in units of number of cases), both averaged across the tracts within each region. Results based on the 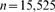 geocoded Maryland Prostate cancer data split into the 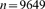 experimental geocodes and 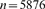 experimental nongeocodes subsets for evaluation.

## Discussion

A general methodological framework was presented for using imputation to provide information for missing spatial data from nongeocoded addresses from a GIS. This framework could be applied in an analytical setting. Three strategies were evaluated, each based on increasing use of available information. Strategy 3, based on the weighted selection of the within zip code age race distribution, performed the best overall in comparison to the other more simplified strategies.

Motivation was provided to have evaluation based not at the individual case level focused on geocoding accuracy but in regards to a substantive outcome of interest that uses location information. Multiple imputation is also employed to assess the uncertainty due to imputation since results based on imputation alone, especially when linking imputed locations to other spatial data, may be questionable. A benefit from this approach worth highlighting in the application here is that users can report not just enumerations based on the total number of successfully geocoded addresses (which could certainly be biased and under-reported), but also include a statistically adjusted count (imputed count) with a measure of uncertainty that are based on all the case data, the geocodes and imputed nongeocodes. Both these features, evaluation based on a relevant analysis outcome and the development of a measure of imputation uncertainty, extends previously cited work in this area [11], [25], [33]. Similar strategies can be applied for the analysis of outcomes other than case enumerations.

Although county-level results were presented, these may not be as interesting as those for some of the smaller Census units due to the fact that (a) the known zip codes are already at a spatial scale which on average is geographically smaller than county and does not require geocoding, and (b) some registries and other data collection entities may have other means for determining county designations that do not require geocoding as was the case with the Maryland prostate cancer data. Furthermore, zip code boundaries only cross a few counties and some counties may completely encompass a single zip code, both of which can markedly increase imputation accuracy. This was evident in Table 1 with several imputed totals very close to the true value, and some with no error using the 95% imputation interval. Contrary to this, the larger number of nongeocodes in need of imputation due to the relative size of counties may impart some inaccuracy due to imputation to adjacent counties. This was also evident with two counties in the Baltimore region in Table 1. The county level did however serve well to demonstrate the process and provide a benchmark for further comparisons.

To demonstrate our imputation based approach and evaluation for nongeocoded addresses only cases with complete age, race, grade, and stage data were used, and focused on white and black cases only, in order to have sufficient sample size in smaller geographic areas to test the methods. The proposed imputation strategies could certainly be applied to cases missing grade or stage and even missing age or race; for the later imputation weights (for Strategy 3) would just be based on the overall population weights. Similar strategies could also be devised for cases without complete zip codes, where perhaps only city or county is known. Also, although the procedures presented are based on data from the United States, exploiting the various Census demographic and boundary data, similar imputation strategies can be devised to accommodate the availability of population data and geographic units from other countries with similar hierarchical geographic, political, and enumeration units.

Opportunities certainly exist to develop more sophisticated imputation strategies with alternative methods of evaluation. As was restated here it is well known that nongeocoded data are often not geographically uniform and commonly a function of population density with rural areas being more susceptible. However, this is certainly not true everywhere all the time. Many factors can contribute to an address not geocoding successfully. New home developments are one example that cause existing base maps to be out of date and hence associated addresses to be nongeocodable but likely located in more urban settings. More relevant to the prostate cancer application and evaluation of the imputation strategies would be the development of new senior group housing, assisted living, and nursing homes. Even for existing institutional cancer cases, geocoding is less than straightforward based on the address reported for these cases and how the Census tabulates institutional populations. The data subset used here is believed to have been more likely to have excluded men whose address of record at the time of diagnosis was a facility as well as a good number of the oldest of cases. Reconciling these types of scenarios prior to imputing is sensible advice [41].

Further, imputation strategies based only on outcome population at risk, such as with our age and race weighted selection Strategy 3, would impart a tendency for imputed case locations to be spatially close to those that geocoded successfully. Against this, the existence of a successfully geocoded case at a particular location would make it less likely that a nongeocoded case (that needs to be imputed) would be nearby. This may explain the results in Table 3 that the percentage of times the 95% imputation interval covered the true totals was higher for Strategy 2 than for Strategy 3 in two of the more rural regions in Maryland, Southern Maryland and the Eastern Shore regions. In a rural zip code with farms surrounding a central area where everyone has mail delivery, the geocoded cases will tend to be in the central area while the nongeocoded cases will tend to be out in the farmland. In such a situation Strategy 2 might be expected to perform better. Disentangling these contrary effects would need attention in the development of more sophisticated imputation methods.

Situations may also arise where the at risk population density does not correlate well with the spatial distribution of the outcome under investigation. In such scenarios distributing the nongeocoded cases along population at risk patterns may overlook underlying unusual distribution of cases and hence potentially lead to an under-detection of such patterns. Alternatives to Strategies 2 and 3 then might be to somehow include the outcome variable which like age and race is likely known for the nongeocoded records or other auxiliary variables (risk factors) that correlate with the spatial distribution of the outcome under investigation. However, in the absence of any such knowledge the more conservative approach towards imputation for nongeocoded cases based only on at risk population density would be less likely to induce bias, but may fail to detect existing patterns unrelated to population density. Future imputation strategies might also consider making use of textual information in the address field to better inform the weighting scheme. Even when records do not geocode, it is often possible at least to identify the correct road, which limits the set of possible imputed locations.

There is often much information associated with nongeocoded records, making their exclusion from follow up analysis a potential concern when done precipitously. This often includes the postal zip code as well as detailed demographic and diagnostic information valuable in epidemiologic applications. From a statistical viewpoint the imputation approach for dealing with nongeocoded data is based on completing the data via imputation (geocoded records plus imputed nongeocoded records) then applying statistical methods to this complete data. The complementary approach of multiple imputation can then be applied to assess uncertainty due to the imputation process. In an alternative approach Zimmerman 2008 [42] proposes a stochastic mechanism to represent the geocoding process and applies this to estimation of the spatial intensity function in point process applications with incomplete geocoding.

There are other limitations and sources of potential bias regarding the geocoding process that warrant mention. In our strategies we assumed not only that the reported zip codes were accurate but also those that geocoded successfully did so correctly. This is certainly not always true even for those with a GIS match score of 100%. Furthermore, the design of our geocoding process was not meant to yield point locations exactly where those addresses actually exist, but rather to approximate between base map road segments using address numbers. Proposals to geocoding that incorporate aerial photography are an alternative [43]. These types of geocoding inaccuracies and/or bias can influence analytic results and depend on the level of spatial resolution required and the statistical methods being applied. These remain active areas for further research [43]–[50].
